# Evaluation of a surgical supervision model in three African countries—protocol for a prospective mixed-methods controlled pilot trial

**DOI:** 10.1186/s40814-019-0409-6

**Published:** 2019-02-18

**Authors:** Chiara Pittalis, Ruairi Brugha, Gloria Crispino, Leon Bijlmakers, Gerald Mwapasa, Chris Lavy, Grace Le, Mweene Cheelo, John Kachimba, Eric Borgstein, Nyengo Mkandawire, Adinan Juma, Paul Marealle, Kondo Chilonga, Jakub Gajewski

**Affiliations:** 1 Department of Epidemiology and Public Health Medicine, Royal College of Surgeons in Ireland, Beaux Lane House, Dublin 2, Ireland; 2 Statistica Medica, 26 Belarmine Court Enniskerry Road Stepaside, Dublin 18, Ireland; 3 Radboud University Medical Centre, Geert Grooteplein Zuid 10, 6525 Nijmegen, GA Netherlands; 4 University of Malawi, College of Medicine, Mahatma Gandhi, Blantyre, Malawi; 5 Nuffield Department of Orthopaedics, Rheumatology and Musculoskeletal Sciences, Nuffield Orthopaedic Centre, University of Oxford, Windmill Road, Oxford, OX3 7HE UK; 6 Surgical Society of Zambia, Department of Surgery, University Teaching Hospital, Nationalist Road, Lusaka, Zambia; 7 ECSA Health Community Secretariat, 157 Olorien, Njiro Road, PO Box 1009, Arusha, Tanzania; 8 Tanzania Surgical Association, P.O. Box 65098, Dar Es Salaam, Tanzania; 9 Kilimanjaro Christian Medical Centre, PO Box 3010, Moshi, Tanzania; 10 Institute of Global Surgery, Royal College of Surgeons in Ireland, 123 St Stephen’s Green, Dublin 2, Ireland

**Keywords:** Surgery, Non-physician clinicians, Surgical training, Supervision, Evaluation, Africa

## Abstract

**Background:**

District-level hospitals (DLHs) can play an important role in the delivery of essential surgical services for rural populations in sub-Saharan Africa if adequately prepared and supported. This article describes the protocol for the evaluation of the Scaling up Safe Surgery for District and Rural Populations in Africa (SURG-Africa) project which aims to strengthen the capacity in district-level hospitals (DLHs) in Malawi, Tanzania and Zambia to deliver safe, quality surgery. The intervention comprises a programme of quarterly supervisory visits to surgically active district-level hospitals by specialists from referral hospitals and the establishment of a mobile phone-based consultation network. The overall objective is to test and refine the model with a view to scaling up to national level.

**Methods:**

This mixed-methods controlled pilot trial will test the feasibility of the proposed supervision model in making quality-assured surgery available at DLHs. Firstly, the study will conduct a quantitative assessment of surgical service delivery at district facilities, looking at hospital preparedness, capacity and productivity, and how these are affected by the intervention. Secondly, the study will monitor changes in referral patterns from DLHs to a higher level of care as a result of the intervention. Data on utilisation of the mobile based-support network will also be collected. The analysis will compare changes over time and between intervention and control hospitals. The third element of the study will involve a qualitative assessment to obtain a better understanding of the functionality of DLH surgical systems and how these have been influenced by the intervention. It will also provide further information on feasibility, impact and sustainability of the supervision model.

**Discussion:**

We seek to test a model of district-level capacity building through regular supervision by specialists and mobile phone technology-supported consultations to make safe surgical services more accessible, equitable and sustainable for rural populations in the target countries. The results of this study will provide robust evidence to inform and guide local actors in the national scale-up of the supervision model. Lessons learned will be transferred to the wider region.

## Background

Conditions amenable to surgery account for an estimated 11–15% of the global disease burden [1], with 1.4 million preventable deaths occurring annually [2]. Yet, access to surgical care around the world continues to be inequitable, particularly in sub-Saharan African (SSA) countries where the performance of health systems is undermined by chronic resource scarcity and severe surgical workforce shortages [3]. Alkire et al. [4] estimated that up to 95% of the population in SSA has limited access to safe, affordable surgical and anaesthetic care because general surgical services are concentrated around urban centres. If comprehensive universal health coverage is to be achieved [5], the surgical needs of rural dwellers need to be met.

District-level hospitals (DLHs) are the first and often the final point of contact for complex, curative health services in rural SSA and, as such, can play an important role in the delivery of essential elective and emergency surgery, as emphasised by the Lancet Commission on Global Surgery [6]. Evidence of safety, feasibility and cost-effectiveness of the provision of surgical services by DLHs is supported by a growing body of empirical research [7–12]. A key challenge, however, is the shortage of qualified staff [13]—surgical clinicians, anaesthetists and nurses—aggravated by labour migration [14], with as few as one surgeon per 2.5 million people in some SSA rural settings [15]. This human resource gap is often filled by health professionals with non-specialist training such as medical officers (MOs), non-physician clinicians (NPCs) [16] and general nurses [17]. Historically, these cadres have been the backbone of clinical care services at first-level facilities in SSA and experiences from a number of countries demonstrate the benefits of allocating surgical responsibilities (i.e. task-shifting) to non-specialists [18–21].

In some countries, there are reservations about non-specialists, especially NPCs, undertaking major surgery [22, 23]. However, it is the lack of support and supervision by specialist surgeons of district-level hospitals staff that precludes the promotion of surgical task shifting and the provision of safe elective and life-saving emergency surgery at DLHs to serve rural populations [17, 23]. The Lancet Commission describes surgical clinicians at DLHs as ‘true generalists’ [6], able to contribute to increasing access to surgery for otherwise neglected rural communities. Capacity building, clinical mentoring and supervision are essential to ensure that non-specialists, both MOs and surgically trained NPCs, are prepared, motivated and supported to deliver safe, quality surgery [2, 6, 24].

### The need for capacity building

Surgery is a complex intervention that requires multi-professional teams to deliver quality-assured surgical, anaesthesia and post-operative nursing care [25], with the additional challenge in DLHs of staff working in isolated and poorly resourced settings. Currently, formal education of MOs, NPCs and general nurses tends to be centralised and highly didactic, with doubts over the effectiveness of this pedagogical model for providing non-specialists with the practical skills needed for the specific challenges of the district hospital [17, 26, 27]. Moreover, in the case of NPCs, there are disparities in training, expertise and job profiles across, even within, countries [16, 26]. The importance of on-the-job training and recognition of NPCs’ competencies by higher level staff, as highlighted in a recent study [17], can impact on staff motivation and quality of surgical care. Opportunities for continuing education and in-service training are limited in DLHs—more so for NPCs than for MOs—which means skills are largely acquired through informal on-the-job exposure [17].

Investment in supervision, mentoring and in-service training could be a viable solution to ensuring safe surgery is delivered at DLHs [17] because (1) it builds capacity without diverting human resources from the workplace or disrupting service delivery, (2) it addresses practical problems in surgery that are reflective of local circumstances, (3) it may be more cost-effective than off-site training and (4) it may motivate and help maintain a stable trained rural workforce. A growing body of evidence demonstrates that the training, supervision and mentoring of NPCs is acceptable, encouraged [28], effective [29] and safe [19, 30, 31]. There are examples of NGO and donor-sponsored visiting surgeons programmes in rural parts of Africa [32, 33]; however, there is a dearth of methodical evaluations. Where evidence exists, it is limited to documenting the outputs of surgical outreach campaigns rather than the value of the supervision delivered by the visiting specialists [34]. Therefore, robust research is required to understand the benefits, or otherwise, of building the surgical capacity of non-specialists through regular supervision, integrated into local health systems, and how to ensure sustainability [35].

## The SURG-Africa study

In 2017, the Scaling up Safe Surgery for District and Rural Populations in Africa (SURG-Africa) project began working with the ministries of health (MoH) in Zambia, Malawi and Tanzania to develop a supervision intervention to improve the delivery of accessible elective and emergency surgery at DLHs, based on the lessons from an earlier initiative in Malawi and Zambia: the Clinical Officer Surgical Training for Africa (COST-Africa) research project [36]. SURG-Africa aims to design, implement and evaluate country-specific models of regular supervisory visits, in-service training and mentoring support by specialists of surgically active clinicians (NPCs, MOs, anaesthetists and theatre nurses), thereby building essential competencies for district surgery. The overall objective is to test and refine the model through a cross-country controlled pilot trial with a view to scaling up to the national level. The purpose is to make safe surgical services accessible, equitable and sustainable in the target countries and to transfer lessons learned to the wider region. This article reports the protocol of this pilot trial to determine the feasibility of implementing the SURG-Africa supervision model and its acceptability to practitioners.

In most sub-Saharan African countries, routine surgical data are not reported or are not reliable [37]. Alkire et al. [4] modelled access to surgery in 180 countries on the basis of four criteria: timeliness, surgical capacity, safety and affordability, and estimated that a large share of the population in LMICs has inadequate access to surgical care. While modelling of access is useful, it is no substitute for primary empirical data on surgical service delivery and the rigorous evaluation of interventions to improve availability [38]. Good (ideally routine) data, if transformed into useful information, will improve decision-making and enable scarce resources to be allocated to support tailored strategies [39]. By evaluating the SURG-Africa intervention, our study aims to provide policymakers with reliable data and evidence to support decision-making, leading ultimately to better health services [40].

### The intervention

The intervention, which was designed and refined during 2017 with local ministries of health and surgical specialists, consists of (1) a programme of quarterly visits to surgically active district-level hospitals by a Surgical Oversight Team (SOT) and (2) the establishment of a surgical network supported by local mobile phone technologies, enabling real-time consultations and establishment of closed referral feedback loops between DLH surgical clinicians and supervisors at referral hospitals (RHs). The members of the SOT include general surgery, anaesthesia and theatre nursing specialists, with other specialists (orthopaedic surgeons or gynaecologists) joining the team if needed. The main components of the proposed model were developed and piloted in the earlier COST-Africa project [17], and—based on lessons learned—have been adapted in SURG-Africa as follows:

- The project employs a participatory action research (PAR) approach [41] to intervention design based on the principle of ‘letting demand emerge’, thus maximising relevance and ownership by beneficiaries
- A holistic approach to capacity building to involve all key members of the core surgical system team (i.e. GMOs, NPCs, nurse anaesthetists, OT and post-operative nurses and other relevant staff) rather than only surgical providers
- Capacity-building activities are tailored to local needs and the potential of each DLH to safely scale-up surgical services
- A reliance on local resources, i.e. specialists from the main RH in each intervention area, to promote sustainable collaborations between DLHs and higher level RHs

## Research objectives

The specific objectives of this research study are as follows:

- To determine whether the intervention is able to affect change in surgical activity at district level. We will assess whether supervision and mentoring will enhance the capacity of district surgical teams, leading to changes in surgical activity. In particular, we expect to observe (1) an increase in the number of procedures regularly performed by the participating hospitals compared to surgical activity before the intervention, as more district clinicians will be trained in handling general surgical procedures and (2) a change in the range of surgical cases done in DLHs as new procedures will be introduced and performed by local teams, where the SOT deems this to be appropriate to local needs, staff skills and resources
- To establish the feasibility of the intervention to improving skills of local surgical teams (including surgery, anaesthesia and nursing staff) as individuals and as a group. We will test whether the support offered through supervision and mentoring will change confidence (as a proxy measure of skills) and preparedness of local teams in performing essential surgical procedures
- To monitor whether the intervention can change referral practices, particularly whether it can reduce the number of unnecessary surgical cases referred by district hospitals compared to the number before the intervention because skills and confidence of DLHs clinicians are expected to improve
- To determine the feasibility and acceptability of the remote consultation mechanism established by the intervention and to assess any changes in communication between DLHs and RHs and in clinical decision-making. The project’s mobile phone-based communication network will give DLH clinicians access to specialist opinion at RHs and will allow real-time consultations with RHs clinicians. The communication network will also facilitate timely feedback to district teams on referred cases

In parallel, the research will investigate process-related aspects of the intervention to determine feasibility and replicability of the proposed regular supervision model, particularly in light of the shortage of specialists at referral institutions and limited budgets. It will identify obstacles and enablers to DLHs supervision done by specialists located at RHs, to build an understanding of what processes need to be put in place for the supervision to be successful in achieving the above objectives.

## Methods

### Study design

**Fig. 1:**
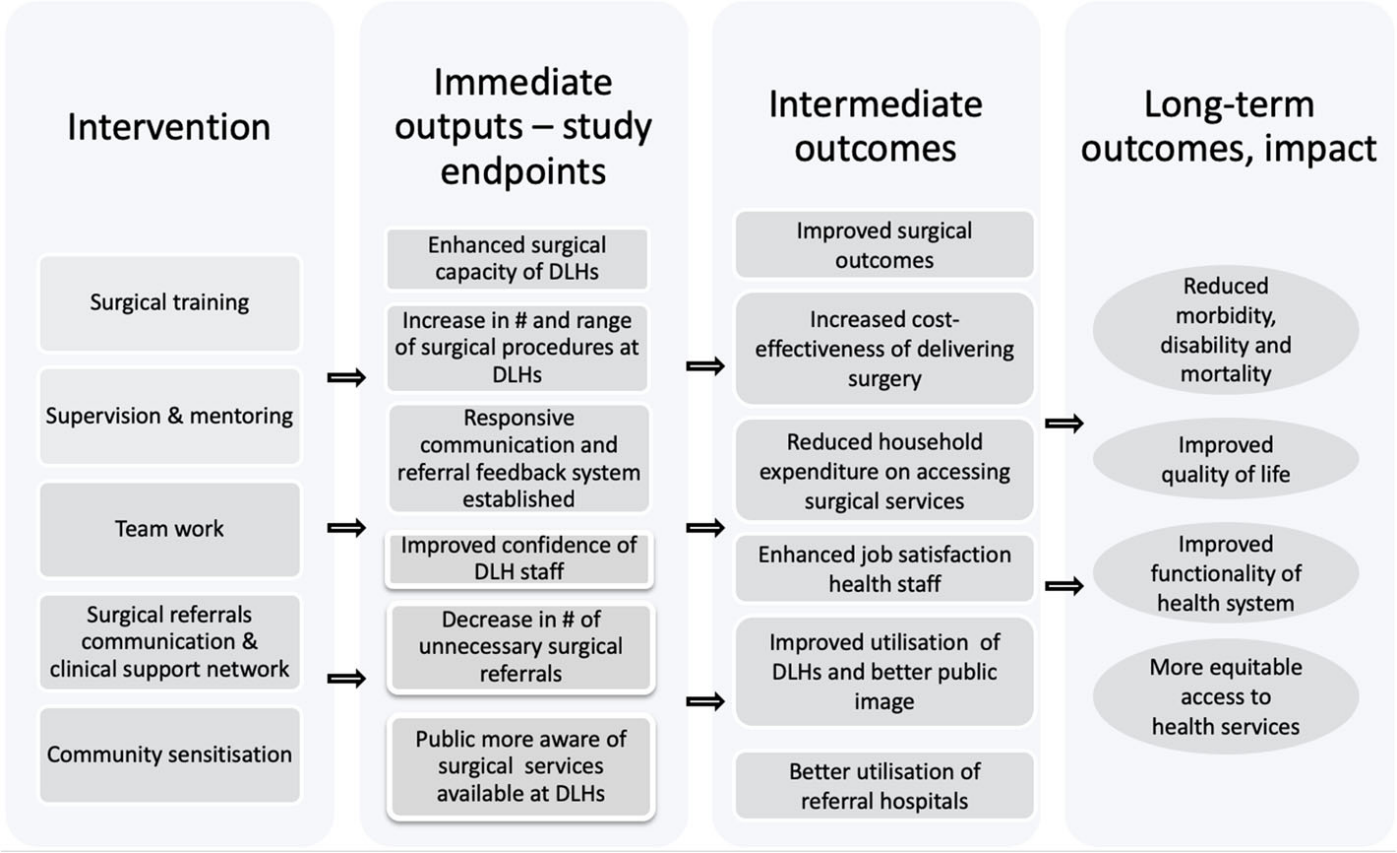
Conceptual framework

This study is a multi-centre, mixed-methods, controlled trial with repeated measures [42] to assess the feasibility of the SURG-Africa intervention, testing new approaches to district hospital supervision. The conceptual framework behind the intervention is illustrated in Fig. 1 below. The rationale behind the study is that building the capacities of district hospitals through supervision, mentoring and training will enable them to provide better services to local populations and avoid unnecessary referrals to higher levels of care, leading to better use of health care resources.

Considering the relatively short life of the project (to 2020), the evaluation will focus primarily on short- and medium-term outputs (second and third column in Fig. 1) as it is not realistic firstly to observe, and secondly to measure, an impact on population-based indicators within this timeframe. Nevertheless, since enhanced functionality of DLHs is expected to lead to improve outcomes of surgery, we anticipate that the intervention should, in the longer perspective, make a positive contribution to the overall health of the populations residing in the areas where participating hospitals are located [43].

The first underlying assumption is that regular supervision of surgery has not been taking place prior to the intervention [6], although every country has ministry of health (MoH) policies in place for this, indicating the importance of regular supervision of DL clinicians. The second one is that the quality of basic surgery, when undertaken by trained surgical clinicians including NPCs, is comparable between the districts and higher levels of care. Previous studies conducted by the research team have already produced empirical evidence on comparatively low surgical complication rates (in preparation) and established that there was no difference in post-operative quality of life of patients who underwent hernia repairs by surgically trained NPCs at district versus specialist hospitals [11]. Similar studies will be conducted by SURG-Africa for a wider set of surgical interventions, with larger sample sizes and in different country settings.

**Fig. 2:**
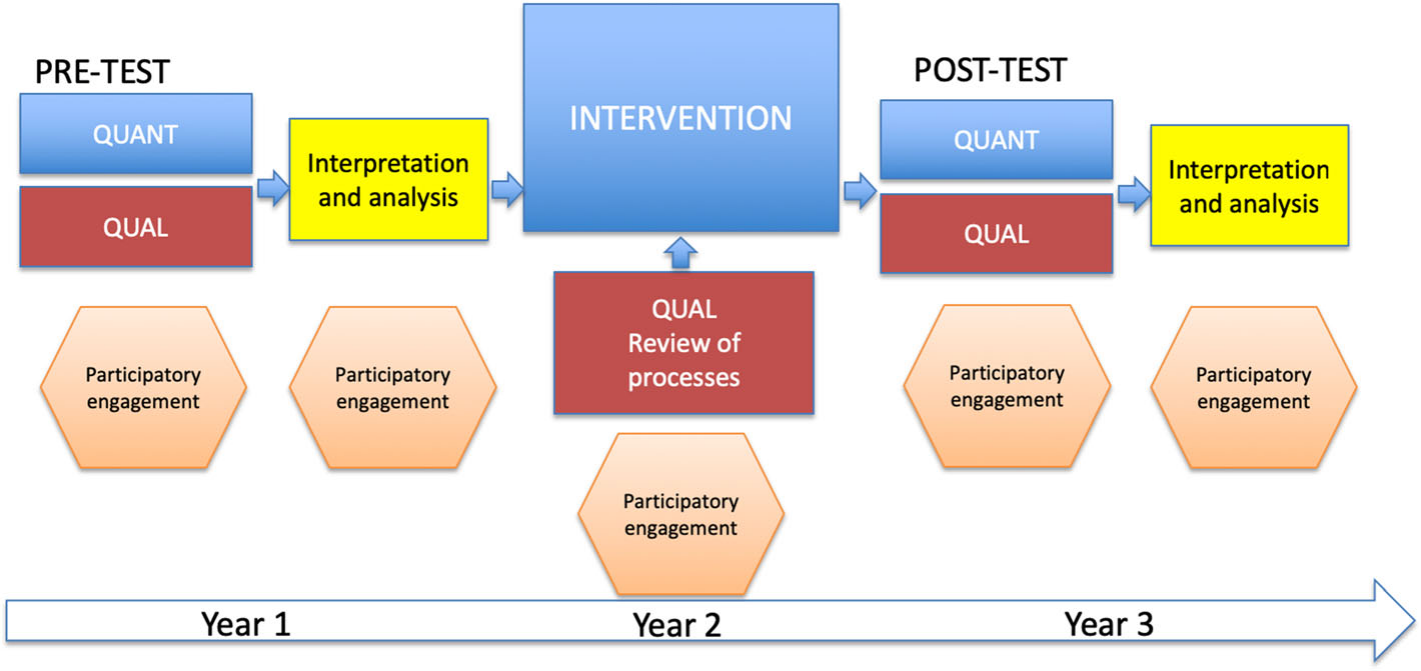
Study structure

The study employs mixed methods, based on a participatory action research (PAR) approach [44–47]. Due to the lack of studies done in the field of district-level supervision, our work is of an exploratory nature [48]. The structure of the study is illustrated in Fig. 2 below.

The description of the trial reported in this paper follows the guidelines provided by the Consolidated Standards of Reporting Trials (CONSORT) statement and its extension for pilot and feasibility trials [49].

### Research sites

The intervention will be delivered in 30 district-level hospitals (DLHs) in three African countries—Malawi, Zambia and Tanzania—for 24 months. The evaluation will include 4 months of baseline data and 24 months of intervention data. Malawi, Zambia and Tanzania were chosen because of their political stability, long-term presence of the consortium members on the ground and logistical considerations related to feasibility of implementation. Malawi and Zambia were the settings of the previous COST-Africa project, whose findings led to the SURG-Africa intervention. In each country, a large catchment population was purposively selected in consultation with local ministries of health, the geographical focus is country-wide in Malawi; adjoining districts in Southern, Central, Western and Lusaka provinces in Zambia and the Northern Zone in Tanzania. Study sites (selected districts) will be determined by the location of referral institutions and respective DLHs that fall within the referral network of that referral hospital. A randomised design for the allocation of hospitals to the intervention and the control arms is not possible because of the small sample sizes (availability of suitable DLHs within these referral networks) and the nature of the intervention (described below). Control hospitals will therefore be located outside these areas and will refer patients to different referral hospitals. However, the risk of selection bias is low because DLHs are fairly homogeneous within each study country regardless of their location, thus reducing the chance of imbalance in characteristics in the distribution of treatment versus control groups [50–52]. Hospitals that do not meet the inclusion criteria (below) or that are already demonstrating significantly better surgical performance than would be expected at a DLH will be excluded from the sample. The precise selection of control hospitals will be determined by the results of the situation analysis conducted prior to the kick off of the intervention. The situation analysis will constitute the baseline for our study (month 0). It will collect information about the surgical capacity of district hospitals using theatre records (volume and range of surgery), Personnel, Infrastructure, Procedures and Supplies (PIPES) scores and additional questions about referral patterns, information management systems, quality control practices and availability of staff (NPCs) not covered by the PIPES questionnaire. This information will be analysed aiming to determine hospital suitability to be part of the intervention based on the following criteria.

Inclusion criteria:

- Capacity to deliver Bellwether surgery: caesarean sections, laparotomy and treatment of open fracture [53]
- Accessibility by car
- Location within the designated referral network in each country, with Livingstone Central Hospital or University Teaching Hospital in Zambia, Queen Elizabeth Central Hospital in Malawi, and Kilimanjaro Christian Medical Centre in Tanzania as focal points for referrals

Exclusion criteria:

- No surgical capacity to deliver Bellwether surgery
- No accessibility by car
- Relatively high surgical capacity (to be determined by an average Personnel, Infrastructure, Procedures, Equipment and Supplies (PIPES) index score—see Table 2 [54])
- Having any specialists (i.e. trained surgeons or anaesthesiologists)

### Intervention and control hospitals

Intervention and control groups will be purposively determined, based on the inclusion/exclusion criteria described above using the results of the baseline situation analysis. The general selection criteria are presented in the table below (Table 1).

**Table 1:** District hospitals selection criteria by country

Country	Intervention	Control
Malawi	All surgically active government-owned district hospitals in the Southern Region	All surgically active hospitals in the Central and Northern Region will be considered for inclusion based on the inclusion/exclusion criteria
Zambia	All surgically active district hospitals in the Southern Province	All surgically active hospitals in Eastern, Western, Lusaka and Central Provinces that have similar scores on the PIPES tool to the scores in the intervention hospitals
Tanzania	All hospitals located in Arusha and Kilimanjaro region of the Northern Zone within a radius of 200 km from KCMC will be considered for the intervention group (similar distance to DLHs that is the furthest in Malawi and Zambia)	Suitable hospitals will be selected based on PIPES score from facilities located in Manyara, Tanga and Singida regions

#### Intervention hospitals

Intervention district hospitals will receive regular visits from the SURG-Africa SOT. Supervisors will be tasked with attending surgical outpatient clinics, conducting ward rounds with DLH surgical clinicians, reviewing theatre management practices, delivering in-service training (including hands-on supervision of elective surgical cases done by the local surgical teams), reviewing post-operative cases and liaising with hospital management to facilitate improvements in surgical care delivery. The in-service training will focus on building the necessary skills and attitudes to deliver safe surgery, appropriate to the district level of care, including among others:

- Clinical decision-making, when to operate, when to observe and when and how to refer the surgical patient to the next level of care (regional or zonal hospital)
- Safe anaesthesia, peri- and post-operative monitoring of surgical patients
- Safe surgery for elective and common emergency conditions appropriate at district level
- Audit and quality assurance processes

Quarterly hospital visits will last two full days, with schedules determined at PAR workshops that bring together key stakeholders (i.e. DLH staff, specialists at referral hospitals, representatives of professional associations and ministries of health) in each of the target countries. Planning of activities will be agreed jointly between SOT and DLH staff ahead of each visit. The aim is to develop a bespoke intervention in each country, addressing the specific local needs of each participating hospital through a series of consultations between DLH staff and their supervisors. Additionally, specialists will provide on-call support to district-level surgical staff from the designated intervention hospitals as needed (e.g. in case of uncertainty about diagnosis, clinical aspects of individual patients requiring surgery, operative and referral decisions, etc.) through a mobile phone-based network.

The SOT will be actively encouraged to provide feedback and engage in constructive exchanges with the trainees during group activities as well as one-on-one mentoring sessions. This mode of delivery is in line with recent studies favouring supportive types of supervision [55] and mentoring [56] over traditional approaches, which often tend to impart knowledge through top-down methods prioritising compliance rather than an engaging type of learning. These ‘rigid’ approaches are seen as less effective and potentially disempowering [56], while supportive supervision is believed to build confidence and promote a positive working environment. All specialists selected for the SOTs will be senior professionals with previous training experience (e.g. in providing post-graduate training at national or specialist hospitals etc.). Through two-day ‘train the trainer’ workshops, SOT members will be trained in supportive supervision methods, quality assurance activities and research data collection methods before DLH visits start in each country to ensure they have the right skills to deliver the intervention.

#### Control hospitals

At baseline, district hospitals in the control group will have similar characteristics to those of hospitals in the intervention group in terms of surgical productivity, PIPES scores and staffing levels (as determined by the findings of the situation analysis). In control sites, practice will continue as standard during the study, without supervision from the SURG-Africa SOTs. The two groups will be followed prospectively and compared to observe any changes as a result of the intervention.

### Study objectives

Our study aims to collect empirical data on a range of output (and in some cases outcome) measures to provide as comprehensive a picture as possible of district-level surgical care, to establish the benefits of the SURG-Africa intervention and to inform national policies and strategies. The evaluation of complex projects such as SURG-Africa requires a variety of tools and multi-level analyses. In order to provide a systematic overview of our evaluation, we have divided our analysis into three broad inter-related feasibility objectives as described below.

#### Objective 1: Surgical preparedness, capacity and productivity

The first objective of our study is to undertake a quantitative assessment of surgical service delivery at district hospitals and how this is affected by the intervention, considering three dimensions: hospital preparedness, capacity and productivity. Each dimension will be measured using quantitative indicators reflecting our overall research aims and conceptual framework, as described in the previous sections. Table 2 provides an overview of objective 1 including indicators, data sources, instruments and planned analyses.

**Table 2:** Overview of objective 1

	Indicators	Data collection instruments	Measurement
Preparedness - change in surgical capacity of DLH facilities	Change in Personnel Infrastructure Procedures Equipment Supplies (PIPES) index scores	PIPES questionnaire	Measured at baseline with periodic follow-ups (month 0, month 12, month 24 of the intervention)
Capacity - change in skills of DLH staff	Number of intervention DLH staff who are reported as competent in doing index procedures Intervention DLH staff self-reported surgical skills confidence levels	PIPES questionnaire Self-reported surgical confidence tool	Before-and-after controlled comparison
Productivity - increase in surgical outputs and outcomes	Change in number of index surgical procedures performed by DLHs Types and numbers of new procedures introduced into DLHs Change in surgical mortality rates	PIPES questionnaire Monthly data collection from DLHs operating theatre registers	Comparing differences: (1) before and after and (2) between intervention and control hospitals

It is expected that the intervention will change the *surgical productivity* of DLHs in terms of number of common surgical procedures performed and types and numbers of new procedures introduced, which form the primary endpoint of the study. Monthly numbers of Caesarean sections are typically used to assess the volume of major surgery at DLHs. SURG-Africa will monitor this parameter and include a larger set of core general surgical procedures done in DLHs, identified through the initial situation analysis. This will allow us to develop an index of key procedures to compare differences in numbers and types of procedures: (1) before and after and (2) between intervention and control hospitals, at both group and individual hospital level of analysis. In parallel, the evaluation will track intra-hospital mortality rates to monitor possible changes in surgical outcomes as surgical activity is expected to increase.

Our study will also analyse whether the intervention will result in changes in the key drivers of DLH productivity, namely the *capacity* of local surgical teams and overall *preparedness* of the facilities to perform surgery. The change in staff capacity will be measured through a before-and-after controlled comparison of the number of intervention DLH staff who is reported as competent in doing index procedures. Another measure is self-reported surgical confidence as a proxy parameter for surgical skills. This tool has been tested and validated by previous studies, showing positive correlation between confidence of surgical providers and assessment of skills by their supervisors [57].

At facility level, the change in the surgical capacity of sample DLHs will be assessed through the Personnel, Infrastructure, Procedures, Equipment and Supply (PIPES) index [51]. Since the intervention focuses primarily on strengthening the human capital of DLHs rather than their physical resources (which will also be monitored), it is expected that the only change directly attributable to the intervention will be on the procedures component of the PIPES index (i.e. the range of procedures done in the intervention hospitals). However, while the intervention does not explicitly target the elements of the PIPES tool that do not relate to human capacity, the attention brought by the project through identifying and reporting obstacles to surgical service provision in the districts might indirectly affect the surgical system, at the district and higher levels, e.g. shifting of resources and/or budget reallocations, horizontally or from higher to district levels. The evaluation will monitor such indirect changes, quantitatively through repeated PIPES measurements and qualitatively through in-depth interviews with DLH and SOT staff.

The PIPES questionnaire captures binary data on five aspects of surgical preparedness of the sample of facilities, namely procedures offered and the availability of personnel, infrastructure, equipment and supplies. Responses to individual categories will then be combined to compute an overall surgical capacity index score for each facility, which is comparable across hospitals and countries. The PIPES questionnaire will be administered directly by the research team to the DLH surgical staff (including representatives from surgery, anaesthesia and nursing). In order to improve reliability and internal validity [58] researchers will use a consensus approach to the completion of the questionnaire, where two to three key informants at each of the DLHs will be asked to discuss and validate the answers. The tool has been tested and validated [58]; however, so as to address gaps identified by other studies employing this instrument [59], we have added a short annex. This delves deeper into the obstacles and opportunities to surgical service provision flagged by the answers to PIPES, and it captures additional details on factors such as anaesthesia capacity, surgical staff expertise, referrals patterns, information management and quality control practices at facility level, based on lessons learned in the earlier COST-Africa project [17].

The PIPES questionnaire, and its annex, will be administered at regular intervals during the life of the project: at baseline (as part of the initial situation analysis), mid-term (month 12) and in the last year (month 24). The primary comparison to detect changes deriving from the intervention will be baseline versus endline data. For the parameters where no tool was available, we have developed tailored data collection instruments. Information on surgical procedures performed in the project sites as well as patients’ age, sex, condition requiring surgery, duration of hospital stay, immediate outcome and cadres of surgical staff involved, among others, will be gathered through a six-monthly review of DLH operating theatre (OT) registers. Relevant data will be organised into an electronic format to facilitate standardisation of records across hospitals. Staff skills’ levels will be tracked through a self-reported surgical confidence tool, covering surgery, anaesthesia and post-op care.

Comparable data from the PIPES questionnaire and OT registers will be cross-checked for further validation of responses and to minimise errors. The surgical confidence tool will be completed by the trainees themselves, anonymously, indicating only their profession. This will minimise the risk of possible bias in responses. Specialist supervisors will be responsible for distributing the forms during their quarterly visits to the districts and collecting them upon completion. As such, its target sample will only include intervention hospitals. All DL clinicians working with members of the SOT will be assessed.

#### Objective 2: Responsive surgical referrals system

A reliable system for monitoring surgical referrals is a critical part of the surgical information system. It can inform decisions about the need to strengthen surgical skills at particular district hospitals from which large volumes of referrals are received at referral hospitals (RHs). Therefore, it is paramount to ensure its correct functioning. However, the team’s previous study (COST-Africa) identified a lack of systematic measures for monitoring flows of surgical referrals. In order to close this data loop, the second objective of our study will be to undertake a quantitative assessment of numbers of surgical referrals sent to RHs within the centre of identified nexuses comprising a group of all district hospitals referring patients to the same central hospital. These nexuses will serve as entry points for delivering and evaluating the supervisory and mentoring intervention.

To strengthen the referral system, a managed clinical network in the form of mobile communicator groups [60, 61] will be established. This will allow for real-time consultations between DLH clinicians and their surgeon supervisors, will facilitate communication prior to referral and will enable the provision of feedback to DLHs once referred patients have received care at the RHs. Data on utilisation of the network will be collected, complementing the data on types of cases referred. The key elements of the planned analysis are summarised in the following table (Table 3).

**Table 3:** Overview of objective 2

	Indicators	Data collection instruments	Measurement
Referrals	Number of surgical cases referred by DLHs to RHs Type of surgical cases referred by DLHs to higher level hospitals Key drivers of referrals	Referral data collection tool (monthly review of data from surgical referral registers maintained at the main RHs) PIPES questionnaire	Analysis of changes in referral patterns over time and between intervention and control hospitals
Responsive communications and close referral feedback mechanisms between DLHs and RHs	Frequency of communications via managed clinical support network Timeliness of communications via managed clinical support network	A logbook monitoring communication between DLH clinicians and specialist supervisors	Analysis of utilisation of the managed clinical support networks

To track these changes, the study will collect anonymised aggregated data on a monthly basis from surgical referral registers maintained at RHs. Data fields, as established in the previous project, will include referring DLH, patient age and sex, reason for referral (presumptive diagnosis), pre-referral treatment, cadre of referring clinician, final diagnosis at RH, treatment/procedure and immediate outcome. Data collectors, trained in the project standard reporting format, will be stationed at the main RHs.

A tool to assess the appropriateness of surgical referrals received by RHs from district hospitals will be developed in consultation with local experts; it will be piloted and used to assess the numbers of unnecessary referrals. A designated clinical network manager will monitor communications between DLH surgical teams and between DLHs and RHs/surgical specialists for the duration of the study. For each DLH/RH, research measures will include cadre of communicating health professional, type (call/text), content type (text/picture/results of investigations), frequency, response timeliness and feedback received.

#### Objective 3: Qualitative assessment of the intervention: obstacles and enablers

The third element of our study involves a qualitative assessment to obtain a better understanding of the functionality of DLH surgical systems (capacity building, decision-making, staff motivation, communications with higher levels, etc.) and how these have been influenced by the intervention. It will also provide further information on the feasibility and impact of the supervision model implemented by the project and will help inform the future scaling up of the intervention under the leadership of local MoHs.

The analysis will look at, but not be limited to, the following:

- Defining what types of elective and emergency surgery can be delivered at what levels—first referral and specialist hospital, by what staff and under what conditions. Questions include the following: What is the (or is there a) national policy for each level? Are there policies/regulations on what types of cases can be delivered at district hospitals, and can agreement be reached with the national stakeholders—MoH, Medical Council and national surgeons—on what cases can be delivered by district-level clinicians, subject to what conditions—training, supervision, etc.?
- Gaining insight into how health-seeking behaviours, population preferences and patient flows to particular hospitals shape the demand for surgery in district communities
- Determining what are the main obstacles and enablers to surgical service delivery at facility level, and how these can be addressed
- Identifying opportunities to establish more effective referral systems, including key drivers of referrals and any changes/improvements attributable to the intervention (e.g. looking at protocols, communication and information systems for district clinicians to consult with surgeons, transport patients and get feedback from referring hospitals)
- In-depth exploration of the appropriateness and potential sustainability of the proposed model, its impact on work practices, staff confidence, attitudes etc. by investigating participants’ experiences of taking part in the supervision and support network
- Assessing the functionality of the support network, and its responsiveness, by analysing participants’ experiences of the clinical support network

The qualitative part of our study will involve semi-structured interviews and focus group discussions (FGDs) with key informants at district, regional, zonal and national levels. District staff may include district medical/health officers; hospital managers and members of the DLH (district-level hospital) surgical team, comprising NPCs, GMOs and nursing staff, covering surgery, anaesthesia and post-op nursing. As a general guide, three staff members are to be interviewed at each DLH, so the total sample will include at least 30 respondents per country. At regional/zonal level, relevant staff may include medical/health officers, referral hospital managers and members of the SOT, including specialists in surgery, anaesthesia, and nursing at the RHs. FGDs and in-depth interviews will be conducted at two points in the life span of the project. Interviews and focus groups will be conducted by the research team. Respondents will be identified by the district medical officers (DMOs) or Theatre In-Charge, and none of the researchers is directly related to participants. All interviews will be anonymous and responses will be analysed in cumulative form. These measures will minimise the risk of bias in the responses.

While each of the three study objectives is treated as a stand-alone for presentation purposes, in practice, they are closely linked and overlapping, with the qualitative assessment, in particular, cross-cutting the other two. We do not expect change to be homogenous across all indicators, but rather expect that while the intervention may be successful at changing some dimensions of district-level surgical capacity, it may fail to do so on other dimensions. However, the expected result of the intervention is a trend that indicates a positive change in the abilities of DLHs to deliver more surgeries. This trend may not be observed in all facilities receiving the intervention.

### Sample size

The hospitals to be considered for inclusion will be selected from the pool of eligible (i.e. surgically active and accessible) hospitals in each geographical area covered by the research, as follows:

- Malawi: 22 eligible hospitals out of 24 public district hospitals in the country
- Zambia: 27 eligible hospitals out of 99 Level 1 (district) hospitals in the country
- Tanzania: 36 eligible hospitals out of 41 district health facilities in the Northern Zone (comprising of Manyara, Tanga, Singida, Arusha and Kilimanjaro regions)

Study design and sample size are dictated by local realities (i.e. number of hospitals eligible to receive the intervention as well as the limited availability of qualified supervisors who can commit themselves to deliver sufficient numbers of visits). Nevertheless, the study will select as many eligible hospitals as possible for its sample in order to ensure country and cross-country representativeness.

### Data analysis

The analysis plan comprises comparison of the changes from baseline to end of study (after 24 months of the intervention) in primary and secondary endpoints. The analysis will involve descriptive and summary statistics (frequency counts) for the quantitative data and comparisons of means (with the relevant parametric or non-parametric test), where before-and-after changes are expected. Results will be presented with 95% confidence intervals. Significance will be set at 5%, two-tailed. We intend to have multiple time-points at which data will be collected. Repeated measures will be made to analyse trends in the primary and secondary outcomes, making the assumption that any secular trends (government investments, staff deployments, etc.) will occur irrespective of the allocation of hospitals to either intervention or control arms of the trial. However, even if this is not the case, important secular activities and events will be captured through the qualitative study phase. This is particularly important in LMICs where attention to surgery is growing and other externally-driven activities may take place [6]. The qualitative data will be analysed using the ‘top-down’ method of theme identification [62].

## Discussion

Strengthening surgical services at district level is emerging as one of the priorities for LMICs [63]. There is a growing body of research supporting the case for investment in DLHs, building on evidence that essential surgery at DLHs can be delivered safely, cost effectively and is affordable and feasible, even in low resource settings [6, 11]. However, there is still a need for more in-depth studies, firstly, to explore surgical systems as complex adaptive systems [64] and, secondly, to provide solutions on how to modify these systems to improve efficiency. Lessons from a comprehensive review of country implementation strategies for scaling-up health system strengthening interventions revealed that there is no simple solution applicable consistently across countries and that research is a vital part of the process [64]. To address this challenge, global partnerships including academics, researchers, non-governmental organisations and surgical societies have been formed [36] and are working to help local governments to develop, implement and evaluate best practices for scale-up [65].

SURG-Africa is an example of such partnerships. Local partner institutions in Zambia, Malawi and Tanzania lead on the implementation of the intervention, ensuring its alignment with the priorities of ministries of health and maintaining engagement with national stakeholders. The development of the supervisory visits programme is led by DLH clinicians and SOT members to ensure the model is demand-driven and reflective of the differences in skills and training needs across sample facilities. The role of the European partners is primarily technical, with focus on the research and evaluation aspects of the project.

This is a real-life evaluation of an ambitious supervision model in a controlled study. The intervention builds on the lessons from two large-scale initiatives that have trained and supervised non-physician clinicians to deliver essential and emergency surgery in 12 African countries: the Clinical Officer Surgical Training for Africa (COST-Africa) project 2011–16 and the COSECSA Oxford Orthopaedic Link (COOL) project. The key elements of the study protocol, namely the PIPES questionnaire with an annex and the interview guide for the collection of baseline qualitative data, were developed on the basis of lessons learned from the previous projects which showed that the use of simple, non-ambitious tools (e.g. limiting the number of variables) aids feasibility, data accuracy and response rates. The tools were piloted in selected sites in Zambia in July 2017 and further adjusted before full implementation. Similarly, the tools employed to monitor surgical outputs derived from the DLH OT registers (e.g. classification of procedures etc.), and referral patterns were informed by experience from the previous research studies. The evaluation of the SURG-Africa model involves a range of quantitative and qualitative elements to capture as comprehensively as possible the potential changes resulting from the intervention. We expect these changes to go beyond the defined and anticipated outcomes described in this paper, so the qualitative aspect of the research will be the key in identifying and investigating any additional unforeseen changes from the intervention. We are aware that the intervention sites may be affected by staff movements, changes in governance or in any other way that may affect the outcomes of the intervention. Similarly, the control sites may be approached by other projects and receive MoH-led interventions, donations or any other form of support that would increase their surgical capacity. This should not be perceived as a problem as the qualitative aspect of the study aims to unravel why the expected changes did or did not appear in some hospitals. We also aim to document obstacles and enablers to scaling up surgical services in rural hospitals. Such approach aims to provide robust evidence to inform and to guide local actors (surgical societies, ministries of health and donors) in the national scale-up of the SURG-Africa supervision model.

Another important aspect of our research is the use of a PAR approach in the development and refinement of the model. The PAR approach will allow us to build a good understanding of what works and what does not work on the ground; it will facilitate the establishment of a trust-based relationship among collaborators and will ensure a sense of ownership among partners and stakeholders involved in the implementation of the project in the participating countries. SURG-Africa was designed as a *health systems trial*, similar to that described by Sando et al. [66]. Firstly, the intervention is being implemented through public sector systems in Tanzania, Malawi and Zambia, incorporating three large geographical areas in the target countries for testing the solutions to be scaled up under the leadership of local ministries. Secondly, the intervention is being delivered by government-employed members of the SOT, as part of what is supposed to be their routine duties as qualified surgeons. The difference between the proposed SURG-Africa model and the usual activities conducted by surgeons when occasionally visiting DLHs is that, in our model, the emphasis is on capacity building rather than on clearing theatre waiting lists in rural facilities [67]. So-called outreach campaigns organised either by MoHs or donor-funded projects are considered to be effective in achieving their goals; however, evidence of this is limited [68]. Moreover, they cannot be regarded as sustainable means of increasing capacity at district level to deliver surgical services when needed [6, 69], and there is a concern about the ethics of such interventions if led by foreign specialists [70].

The ultimate goal of SURG-Africa is to engage local stakeholders and produce a sustainable solution which is developed, implemented and owned locally. To our best knowledge, there has not been any published study looking at strengthening DLHs surgical capacity, mobilising existing resources through a programme of regular supervisory visits that includes mentorship, supervision and hands-on training for already practicing surgical care providers. SURG-Africa aims to fill this gap. The expected result is an evidence-based supervision model ready for national scale-up in the participating countries, championed by ministries of health. Through dissemination activities, we aim to transfer lessons learned in Malawi, Zambia and Tanzania to other SSA countries interested in investing in rural surgical services.

## Abbreviations

DLH: District-level hospital
MoH: Ministry of Health
NCP: Non-clinician physician
PIPES: Personnel Infrastructure Procedures Equipment Supplies
RH: Referral hospital
SOT: Surgical Oversight Team

## References

[1] Debas HT, Gosselin R, McCord C, Dean TJ (2006). Surgery. Disease control priorities in developing countries.

[2] Luboga S, Macfarlane SB, Von Schreeb J (2009). Increasing access to surgical services in sub-Saharan Africa: priorities for national and international agencies recommended by the Bellagio Essential Surgery Group. PLoS Med.

[3] Ozgediz D, Riviello R (2008). The “other” neglected diseases in global public health: surgical conditions in sub-Saharan Africa. PLoS Med.

[4] Alkire BC, Raykar NP, Shrime MG (2015). Global access to surgical care: a modelling study. Lancet Glob Health.

[5] O’Connell T, Rasanathan K, Chopra M (2014). What does universal health coverage mean?. Lancet.

[6] Meara JG, Leather AJM, Hagander L (2015). Global Surgery 2030: evidence and solutions for achieving health, welfare, and economic development. Lancet.

[7] Sani R. The impact of launching surgery at the district level in Niger. World J Surg. 2009;33. 10.1007/s00268-009-0160-x. 10.1007/s00268-009-0160-x PMC2746305 19653031

[8] Grimes CE, Law RSL, Borgstein ES (2012). Systematic review of met and unmet need of surgical disease in rural sub-Saharan Africa. World J Surg.

[9] McCord C, Kruk ME, Mock CN (2015). Organization of essential services and the role of first-level hospitals. Essential surgery: disease control priorities, 3rd ed.

[10] Grimes CE, Law R, Dare A (2017). Cost-effectiveness of two government district hospitals in sub-Saharan Africa. World J Surg.

[11] Gajewski J, Conroy R, Bijlmakers L (2018). Quality of surgery in Malawi: comparison of patient-reported outcomes after hernia surgery between district and central hospitals. World J Surg.

[12] Cornelissen D, Mwapasa G, Gajewski J (2018). The cost of providing district-level surgery in Malawi. World J Surg.

[13] Hoyler M, Finlayson SRG, McClain CD (2014). Shortage of doctors, shortage of data: a review of the global surgery, obstetrics, and anesthesia workforce literature. World J Surg.

[14] Spiegel DA, Gosselin RA (2007). Surgical services in low-income and middle-income countries. Lancet.

[15] Pollock JD, Love TP, Steffes BC (2011). Is it possible to train surgeons for rural Africa? A report of a successful international program. World J Surg.

[16] Mullan F, Frehywot S (2007). Non-physician clinicians in 47 sub-Saharan African countries. Lancet.

[17] Gajewski J, Mweemba C, Cheelo M (2017). Non-physician clinicians in rural Africa: lessons from the medical licentiate programme in Zambia. Hum Resour Health.

[18] Bolkan HA, van Duinen A, Waalewijn B (2017). Safety, productivity and predicted contribution of a surgical task-sharing programme in Sierra Leone. Br J Surg.

[19] Chilopora G, Pereira C, Kamwendo F (2007). Postoperative outcome of caesarean sections and other major emergency obstetric surgery by clinical officers and medical officers in Malawi. Hum Resour Health.

[20] Dresser C, Periyanayagam U, Dreifuss B (2017). Management and outcomes of acute surgical patients at a district hospital in Uganda with non-physician emergency clinicians. World J Surg.

[21] Wilhelm TJ, Dzimbiri K, Sembereka V (2017). Task-shifting of orthopaedic surgery to non-physician clinicians in Malawi: effective and safe?. Trop Dr.

[22] Hoyler M, Hagander L, Gillies R, et al. Surgical care by non-surgeons in low-income and middle-income countries: a systematic review. Lancet. 2015. 10.1016/S0140-6736(15)60837-6. 10.1016/S0140-6736(15)60837-6 26313091

[23] Wilson A, Lissauer D, Thangaratinam S (2011). A comparison of clinical officers with medical doctors on outcomes of caesarean section in the developing world: meta-analysis of controlled studies. BMJ.

[24] World Health Organization (2007). Task shifting: rational redistribution of tasks among health workforce teams: global recommendations and guidelines.

[25] Singer SJ, Molina G, Li Z, Jiang W (2016). Relationship between operating room teamwork, contextual factors, and safety checklist performance. J Am Coll Surg.

[26] Ashengo T, Skeels A, EJH H (2017). Bridging the human resource gap in surgical and anesthesia care in low- resource countries: a review of the task sharing literature.

[27] Cumbi A, Pereira C, Malalane R, et al. Major surgery delegation to mid-level health practitioners in Mozambique: health professionals’ perceptions. Hum Resour Health. 2007. 10.1186/1478-4491-5-27. 10.1186/1478-4491-5-27 PMC2235883 18062808

[28] Galukande M, Kaggwa S, Sekimpi P (2013). Use of surgical task shifting to scale up essential surgical services: a feasibility analysis at facility level in Uganda. BMC Health Serv Res.

[29] Wilhelm TJ, Thawe IK, Mwatibu B (2011). Efficacy of major general surgery performed by non-physician clinicians at a central hospital in Malawi. Trop Dr.

[30] Chu KM, Ford NP, Trelles M. Providing surgical care in Somalia: a model of task shifting. Confl Health. 2011. 10.1186/1752-1505-5-12. 10.1186/1752-1505-5-12 PMC3152518 21762491

[31] Nyamtema AS, Pemba SK, Mbaruku G, et al. Tanzanian lessons in using non-physician clinicians to scale up comprehensive emergency obstetric care in remote and rural areas. Hum Resour Health. 2011. 10.1186/1478-4491-9-28. 10.1186/1478-4491-9-28 PMC3230124 22071096

[32] Bailey C, Blake C, Schriver M (2016). A systematic review of supportive supervision as a strategy to improve primary healthcare services in sub-Saharan Africa. Int J Gynecol Obstet.

[33] Outreach Programme - AMREF Flying Doctors. https://flydoc.org/outreach-programme/ Accessed 6 May 2018.

[34] Shah JN (2015). Taking specialist surgical services to the rural district hospitals at one forth cost: a sustainable ‘return on investment’ public health initiative of Patan Hospital, Patan Academy of Health Sciences, Nepal. Kathmandu Univ Med J.

[35] Chu K, Rosseel P, Gielis P, et al. Surgical task shifting in sub-Saharan Africa. PLoS Med. 2009. 10.1371/journal.pmed.1000078. 10.1371/journal.pmed.1000078 PMC2677109 19440533

[36] Gajewski J, Bijlmakers L, Brugha R. Global surgery -informing national strategies for scaling up surgery in sub-Saharan Africa. Int J Health Policy Manag. 2018. 10.15171/ijhpm.2018.27. 10.15171/ijhpm.2018.27 PMC6015509 29935124

[37] Uribe-Leitz T, Jaramillo J, Maurer L (2016). Variability in mortality following caesarean delivery, appendectomy, and groin hernia repair in low-income and middle-income countries: a systematic review and analysis of published data. Lancet Glob Health.

[38] Wong EG, Deckelbaum DL, Razek T (2015). Global access to surgical care: moving forward. Lancet Global Heal.

[39] Lippeveld T, Sauerborn R, Bodart C (2000). Design and implementation of health information systems.

[40] Health Metrics Network & World Health Organization (2008). Framework and standards for country health information systems.

[41] Subramanian S, Naimoli J, Matsubayashi T, et al. Do we have the right models for scaling up health services to achieve the Millennium Development Goals? BMC Health Serv Res. 2011. 10.1186/1472-6963-11-336. 10.1186/1472-6963-11-336 PMC3260120 22168915

[42] Cohen E, Creswell JW, Plano Clark VL (2008). Book review. Designing and conducting mixed methods research.

[43] Ozgediz D, Riviello R (2008). The other neglected diseases in global public health: surgical conditions in sub-Saharan Africa. PLoS Med.

[44] Berg BL (2009). Qualitative research methods for the social sciences.

[45] Israel BA, Schulz AJ, Parker EA (1998). Review of community-based research: assessing partnership approaches to improve public health. Annu Rev Public Health.

[46] Wallerstein N, Duran B (2010). Community-based participatory research contributions to intervention research: the intersection of science and practice to improve health equity. Am J Public Health.

[47] Baum F, MacDougall C, Smith D (2006). Participatory action research. J Epidemiol Community Health.

[48] Singh K (2007). Quantitative social research methods.

[49] Eldridge SM, Chan CL, Campbell MJ (2016). CONSORT 2010 statement: extension to randomised pilot and feasibility trials. Pilot Feasibility Stud.

[50] Lavy C, Tindall A, Steinlechner C (2007). Surgery in Malawi–a national survey of activity in rural and urban hospitals. Ann R Coll Surg Engl.

[51] Esquivel MM, Uribe-Leitz T, Makasa E (2016). Mapping disparities in access to safe, timely, and essential surgical care in Zambia. JAMA Surg.

[52] Baker T, Lugazia E, Eriksen J, et al. Emergency and critical care services in Tanzania: a survey of ten hospitals. BMC Health Serv Res. 2013. 10.1186/1472-6963-13-140. 10.1186/1472-6963-13-140 PMC3639070 23590288

[53] O’Neill KM, Greenberg SLM, Cherian M (2016). Bellwether procedures for monitoring and planning essential surgical care in low- and middle-income countries: caesarean delivery, laparotomy, and treatment of open fractures. World J Surg.

[54] Groen RS, Kamara TB, Dixon-Cole R (2012). A tool and index to assess surgical capacity in low income countries: an initial implementation in Sierra Leone. World J Surg.

[55] McAuliffe E, Daly M, Kamwendo F, et al. The critical role of supervision in retaining staff in obstetric services: a three country study. PLoS One. 2013. 10.1371/journal.pone.0058415. 10.1371/journal.pone.0058415 PMC3605440 23555581

[56] Schwerdtle P, Morphet J, Hall H. A scoping review of mentorship of health personnel to improve the quality of health care in low and middle-income countries. Glob Health. 2017. 10.1186/s12992-017-0301-1. 10.1186/s12992-017-0301-1 PMC5627414 28974233

[57] Geoffrion R, Lee T, Singer J (2013). Validating a self-confidence scale for surgical trainees. J Obstet Gynaecol Can.

[58] Markin A, Barbero R, Leow JJ (2014). Inter-rater reliability of the PIPES tool: validation of a surgical capacity index for use in resource-limited settings. World J Surg.

[59] Nwanna-Nzewunwa OC, Ajiko M-M, Kirya F (2016). Barriers and facilitators of surgical care in rural Uganda: a mixed methods study. J Surg Res.

[60] Henry JV, Winters N, Lakati A (2016). Enhancing the supervision of community health workers with WhatsApp mobile messaging: qualitative findings from 2 low-resource settings in Kenya. Glob Health Sci Pract.

[61] Petruzzi M, De Benedittis M (2016). WhatsApp: a telemedicine platform for facilitating remote oral medicine consultation and improving clinical examinations. Oral Surg Oral Med Oral Pathol Oral Radiol.

[62] Boyatzis RE (1998). Transforming qualitative information: thematic analysis and code development.

[63] Price R, Makasa E, Hollands M (2015). World Health Assembly Resolution WHA68.15: ‘strengthening emergency and essential surgical care and anesthesia as a component of universal health coverage’ - addressing the public health gaps arising from lack of safe, affordable and accessible surgical and anesthetic services. World J Surg.

[64] Paina L, Peters DH (2012). Understanding pathways for scaling up health services through the lens of complex adaptive systems. Health Policy Plan.

[65] Peter NA, Pandit H, Le G (2016). Delivering a sustainable trauma management training programme tailored for low-resource settings in east, central and southern African countries using a cascading course model. Injury.

[66] Sando D, Geldsetzer P, Magesa L, et al. Evaluation of a community health worker intervention and the World Health Organization’s Option B versus Option A to improve antenatal care and PMTCT outcomes in Dar es Salaam, Tanzania: study protocol for a cluster-randomized controlled health systems implementation trial. Trials. 2014. 10.1186/1745-6215-15-359. 10.1186/1745-6215-15-359 PMC4247663 25224756

[67] Evans FM, Nabukenya MT (2017). Con: pure service delivery is no longer needed in global surgical missions. Can J Anesth Can d’anesthésie.

[68] Sykes KJ (2014). Short-term medical service trips: a systematic review of the evidence. Am J Public Health.

[69] Welling DR, Ryan JM, Burris DG (2010). Seven sins of humanitarian medicine. World J Surg.

[70] Clarke DL, Aldous C (2013). Surgical outreach in rural South Africa: are we managing to impart surgical skills?. S Afr Med J.

